# Optimization of a murine and human tissue model to recapitulate dermal and pulmonary features of systemic sclerosis

**DOI:** 10.1371/journal.pone.0179917

**Published:** 2017-06-26

**Authors:** Tomoya Watanabe, Tetsuya Nishimoto, Logan Mlakar, Jonathan Heywood, Maya Malaab, Stanley Hoffman, Carol Feghali-Bostwick

**Affiliations:** Division of Rheumatology & Immunology, Department of Medicine, Medical University of South Carolina, Charleston, South Carolina, United States of America; University of Alabama at Birmingham, UNITED STATES

## Abstract

The murine bleomycin (BLM)-induced fibrosis model is the most widely used in systemic sclerosis (SSc) studies. It has been reported that systemic delivery of BLM via continuous diffusion from subcutaneously implanted osmotic minipumps can cause fibrosis of the skin, lungs, and other internal organs. However, the mouse strain, dosage of BLM, administration period, and additional important features differ from one report to the next. In this study, by employing the pump model in C57BL/6J mice, we show a dose-dependent increase in lung fibrosis by day 28 and a transient increase in dermal thickness. Dermal thickness and the level of collagen in skin treated with high-dose BLM was significantly higher than in skin treated with low dose BLM or vehicle. A reduction in the thickness of the adipose layer was noted in both high and low dose groups at earlier time points suggesting that the loss of the fat layer precedes the onset of fibrosis. High-dose BLM also induced dermal fibrosis and increased expression of fibrosis-associated genes *ex vivo* in human skin, thus confirming and extending the *in vivo* findings, and demonstrating that a human organ culture model can be used to assess the effect of BLM on skin. In summary, our findings suggest that the BLM pump model is an attractive model to analyze the underlying mechanisms of fibrosis and test the efficacy of potential therapies. However, the choice of mouse strain, duration of BLM administration and dose must be carefully considered when using this model.

## Introduction

Systemic sclerosis (SSc) is a multisystem connective tissue disease characterized by immune dysregulation, obliterative vasculopathy, and fibrosis of skin and internal organs. It has the highest disease-related mortality and morbidity with an impaired quality of life among the rheumatologic illnesses [1, 2]. Fibrosis is caused by fibroblast activation, proliferation and increased deposition of extracellular matrix (ECM) proteins such as fibronectin and collagen in various organs [3, 4]. Progression of organ fibrosis leads to end-stage organ failure as a result of the loss of normal structure and function. Notably, interstitial lung disease (ILD) is currently one of the most significant complications of SSc. ILD is currently the leading cause of death in patients with SSc [5]. Furthermore, skin fibrosis causes reduction of wrinkles and disappearance of the cutaneous furrows. These features lead to diminished mouth opening and width, concomitance of sicca syndrome, and finger joint contractures with impaired quality of life. However, the mechanisms underlying organ fibrosis remain to be completely elucidated.

The availability of an animal model that mimics the typical features of a human disease is essential for the study of that disease. The bleomycin (BLM)-induced fibrosis model is the most widely used murine model of SSc [6–8]. To induce murine dermal fibrosis, daily subcutaneous (sc) injection of BLM for 4–6 weeks is commonly used and results in local dermal inflammation and fibrosis [9]. However, this model has disadvantages such as localized cutaneous effects, rare involvement of lung fibrosis, and a requirement for repeated local injections. To induce lung fibrosis, intratracheal or oropharyngeal administration is commonly used, but histological examination of murine lung tissues suggests that lung involvement does not closely mimic human SSc-associated ILD.

A few reports have shown that systemic delivery of BLM via continuous diffusion from subcutaneously implanted osmotic minipumps can cause fibrosis of the skin, lungs, and other internal organs [10–12]. This approach may provide the opportunity to test the effects of potential therapeutic treatments on fibrosis in each of the affected organs simultaneously. However, in spite of the availability of four publications using this model, the mouse strain, dosage and source of BLM, and administration period, differ from one study to the next [11–14]. The choice of mouse strain is of particular importance in this model. It was recently reported that numerous C57BL/6 sublines, which are commonly used for the generation and analysis of transgenic and knockout models, were found to carry an undetected mutation that affected the results of immune system research studies [15]. Several C57BL/6 substrains are genetically and phenotypically different because each of these can be purchased from different animal vendors who have maintained separate breeding stocks, allowing genetic differences to accumulate due to individual variability and genetic drift.

In this study, we investigated the effect of systemically administered BLM, with respect to dose-dependent changes in pulmonary fibrosis, dose-and time-dependent changes in dermal involvement, and loss of the subcutaneous adipose layer in C57BL/6J mice using the pump model. We also examined the effect of BLM on human skin maintained in organ culture.

## Material and method

### 1. Mouse model for systemic BLM delivery using osmotic minipumps

Systemic administration of BLM was done as previously described (11) with some modifications. Briefly, 8 week-old male C57BL/6J mice (The Jackson Laboratory, Bar Harbor, ME, USA) were administrated BLM (Hospira Inc., USA) using osmotic minipumps (ALZET1007D; DURECT Corporation, Cupertino, CA) containing either 100μl saline as vehicle or BLM for 7 days. BLM was dissolved in PBS at concentrations of 1.0 U/kg, 10 U/kg, 60 U/kg, and 110 U/kg. On day 0, osmotic minipumps were implanted under the back skin of mice slightly posterior to the scapulae under isoflurane anesthesia. Pumps were removed on day 7. Lung and skin tissues were harvested on days 7, 10, 14, 21, and 28 for histological and gene expression analysis and hydroxyproline assays. Skin samples were obtained from the dorsal area, approximately 2 cm posterior to the pump implantation site, and abdominal area. All experiments were done under a protocol approved by the IACUC of the Medical University of South Carolina (MUSC).

### 2. Histological analysis

Skin and lung specimens were fixed with 10% formalin and embedded in paraffin. Six micrometer sections of paraffin-embedded mouse skin specimens were stained with hematoxylin and eosin (H&E) or Masson’s trichrome (MT). Images were captured on a Motic BA410 Compound Microscope (Motic, British Columbia, Canada) using identical settings.

### 3. Hydroxyproline assay

To quantify the amount of collagen in mouse skin and lung specimens, hydroxyproline content was measured as previously described [16].

### 4. Statistical analysis

All continuous variables were expressed as the mean ± standard deviation. All statistical analyses were done using IBM SPSS statistics 22 (IBM Corporation, NY, USA). Comparison among 3 or more groups was done using ANOVA with post-hoc Tukey’s test to evaluate statistical significance.

### 5. Quantitative PCR

Total RNA was extracted from mouse skin using TRIZOL Lysis Reagent (Life technologies, CA, USA) and RNeasy^®^ kit (Qiagen Inc., CA, USA). Reverse transcription was performed with SuperScript Ⅳ (Invitrogen, CA, USA). Gene mRNA expression levels were evaluated by quantitative PCR using the TaqMan^®^ real-time PCR system (Life Technologies, CA, USA) according to the manufacturer's protocol on a TaqMan^®^ Gene Expression Assays (Step One Plus real time PCR system, Life technologies, CA, USA). Premixed PCR primers and TaqMan probes for mouse Fn, Coll1α1, Tgfb1, Ctgf, and Gapdh and human FN, Coll1α1, Tgfβ1, Ctgf, and Gapdh were obtained from Life Technologies. Gene expression levels were normalized to Gapdh and compared with the 2^−ΔΔCt^ method.

### 6. *Ex vivo* human skin assays

Injection method: Normal human skin was obtained from residual tissue following plastic surgery. All tissues were obtained according to the guidelines of the Medical University of South Carolina without any identifiers. Subcutaneous fat tissue was removed and skin tissue was cut into 1.5 cm x 1.5 cm sections. Skin tissues were injected intradermally as we previously reported [17, 18] with a total volume of 100 μl of 1×PBS: BLM (1 or 10 mU/ml) (Nippon Kayaku, Tokyo, Japan) or 1× PBS as a vehicle control. Skin samples were cultured in an air-liquid interface with the epidermal side up. The culture medium was replaced after 72h and consisted of Dulbecco’s modified Eagle’s medium (DMEM) (Mediatech, VA, USA) supplemented with 10% FBS (Sigma-Aldrich, MO, USA), penicillin, streptomycin, and anti-mycotic agent (Invitrogen Life Technologies, CA, USA). After 7 days, skin tissue corresponding to an area with 8-mm diameter centered around the injection site was harvested with a disposable biopsy 3 mm punch (Integra York PA Inc., PA, USA) and fixed in 10% formalin prior to embedding in paraffin.

Immersion method: Normal human skin was cut with a disposable biopsy 3 mm punch, and the pieces of tissue were cultured in medium containing BLM (1 or 10 mU/ml) or 1× PBS as a vehicle control. The culture medium was replaced at 72h and consisted of DMEM supplemented with penicillin, streptomycin, and anti-mycotic agent. Skin tissues were harvested 48 hours (for RNA) or 7 days (for hydroxyproline) post-treatment.

## Results

### Dose-dependent increase in lung fibrosis but not skin fibrosis

**Fig 1:**
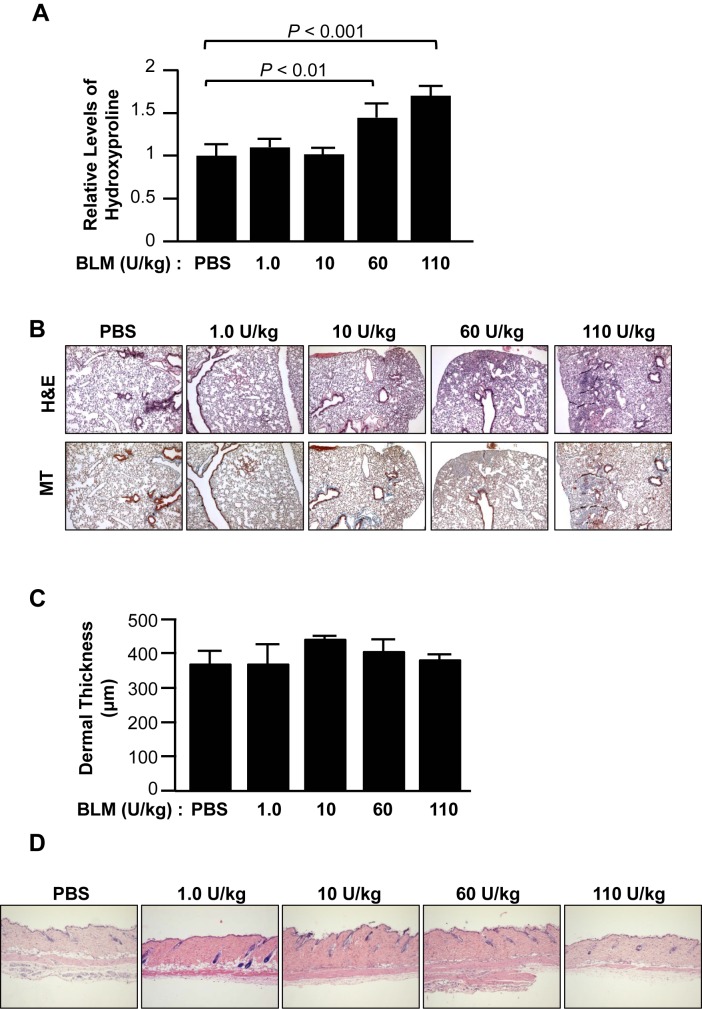
Dose-dependent changes in mouse lung and skin using the pump model. Mice treated with the indicated doses of BLM were sacrificed on day 28. (A) The amount of collagen in the lungs was quantified using hydroxyproline assay (PBS, N = 12; 1.0 U/kg, N = 9; 10 U/kg, N = 5; 60 U/kg, N = 5; 110 U/kg, N = 5). (B) Representative images of hematoxylin and eosin (H&E) (upper) and Masson trichrome (MT) (lower) stained sections of control and BLM–treated lung tissues (original magnification ×40). (C) Dermal thickness was measured in dorsal mouse skin (PBS, N = 16; 1.0 U/kg, N = 5; 10 U/kg, N = 4; 60 U/kg, N = 5; 110 U/kg, N = 5). (D) Representative images of H&E stained sections of BLM–treated skin tissues (original magnification ×40).

To evaluate dose-dependent changes induced by BLM, we measured hyrdoxyproline content and examined the morphology of lung and skin tissues of mice receiving BLM via the pump model. Mice treated with BLM showed a dose-dependent increase in lung fibrosis. Hydroxyproline levels were significantly greater in lungs from mice treated with high dose BLM (60 and 110 U/kg) than those treated with PBS or low dose BLM (1 and 10 U/kg) (Fig 1A) 28 days post-implantation of the pumps. Mice treated with high dose BLM developed subpleural thickening of alveolar walls, narrowed alveolar spaces, and fibrosis (Fig 1B). We further compared time-dependent changes in lung tissues. Inflammatory cells were present in lung treated with a high dose of BLM 4 days following initiation of BLM administration. Inflammatory cell infiltration increased time-dependently in mice treated with high dose BLM compared to control and low dose BLM-treated mice (S1 Fig).

Unlike the lung, skin fibrosis did not increase in a dose-dependent manner (Fig 1C and 1D). This is in contrast to previous reports showing induction of dermal fibrosis within 4 weeks of BLM administration [11–14], suggesting that dermal fibrosis in C57BL/6J mice using the pump model may differ from other mouse strains, and may depend on the dose of BLM and duration of the model.

### Increased skin fibrosis in the early phase of the model

**Fig 2:**
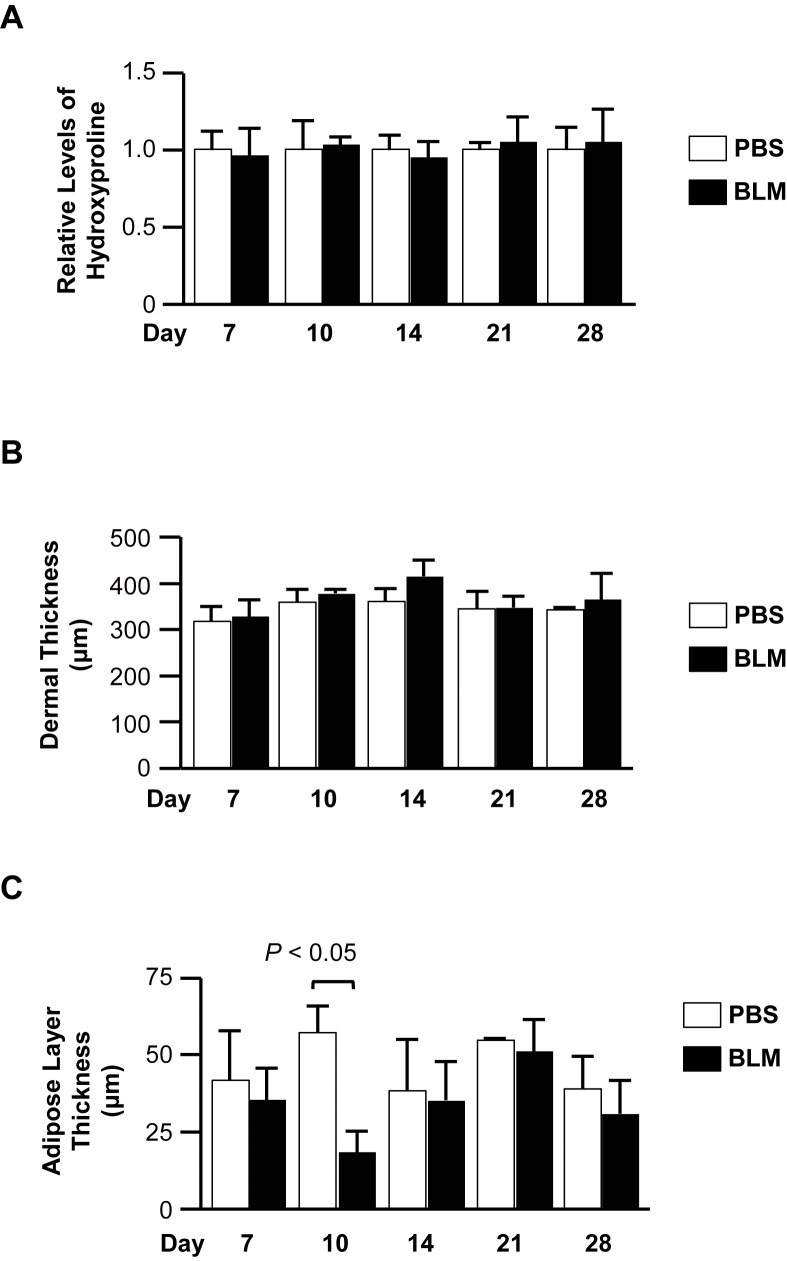
The effect of low dose BLM on dermal and pulmonary fibrosis. Mice treated with BLM (1 U/kg) were sacrificed on days 7 (N = 7), 10 (N = 4), 14 (N = 4), 21 (N = 4), and 28 (N = 4). (A) Hydroxyproline levels were measured in lungs using hydroxyproline assay. (B) Dermal thickness was measured in dorsal skin. (C) Subcutaneous adipose layer thickness was measured in dorsal skin.

We next compared time-dependent changes in dermal fibrosis. Mice treated with a low dose of BLM (1 U/kg) were sacrificed on days 7, 10, 14, 21, and 28. Although neither dermal thickness nor hydroxyproline levels showed any significant changes at all the time points tested (Fig 2A and 2B), the thickness of the subcutaneous adipose layer was significantly decreased in BLM-treated mice compared to control mice on day 10 (Fig 2C). Further, the decrease in adipose layer thickness preceded the trend for increased dermal thickness on day 14 (Fig 2B).

**Fig 3:**
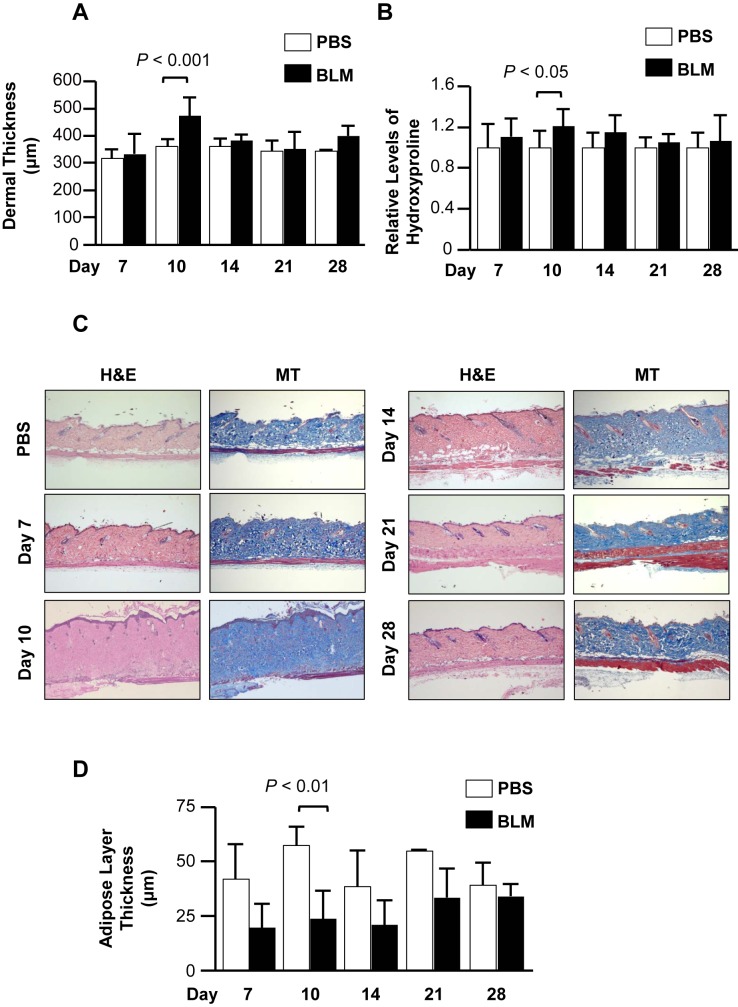
The effects of high dose BLM on dermal fibrosis. Mice treated with BLM (60 U/kg) were sacrificed on days 7 (N = 7), 10 (N = 12), 14 (N = 7), 21 (N = 12), and 28 (N = 6). (A) Dermal thickness was measured in the dorsal skin. (B) Hydroxyproline content was measured in skin tissues shown in (A). (C) Representative H&E (left) and MT (right) images of mouse skin treated with BLM (original magnification ×40). (D) Subcutaneous adipose layer thickness was measured in the dorsal skin.

To compare the effect of different doses of BLM on dermal fibrosis, mice were treated with a high dose of BLM (60 U/kg) and sacrificed on days 7, 10, 14, 21, and 28. On day10 post-BLM treatment (3 days after the removal of the pump), dermal thickness on the back of the mice was significantly increased in BLM-treated mice compared to control mice (Fig 3A–3C). No significant differences in dermal thickness were noted on days 14–28. Hydroxyproline confirmed results obtained with dermal thickness measurement and revealed that the amount of collagen on day 10 was significantly higher in BLM-treated mouse skin than in the skin of control mice (Fig 3D). However, on days 14, 21, and 28 post-BLM treatment, there was no significant change in dermal thickness or hydroxyproline levels. This result indicated that skin fibrosis is induced in the early phase post-bleomycin, but decreases with time. In addition, the thickness of the subcutaneous adipose layer was significantly decreased in BLM-treated mice on days 7 & 10 post-BLM treatment (Fig 3B and 3C). These results suggest that BLM induced an increase of dermal fibrosis that partially replaced the subcutaneous fat. Our results further suggest that a significant loss in the thickness of the fat layer precedes the significant increase in dermal thickness and hydroxyproline levels. To assess whether dermal fibrosis extends beyond the skin on the back, we also evaluated abdominal skin thickness. Unlike the back skin, there was no significant change in abdominal skin thickness for the duration of the experiment (S2A and S2B Fig). This observation indicates that the extent of dermal fibrosis using the BLM pump model may depend on the distance from the pump implantation site.

**Fig 4:**
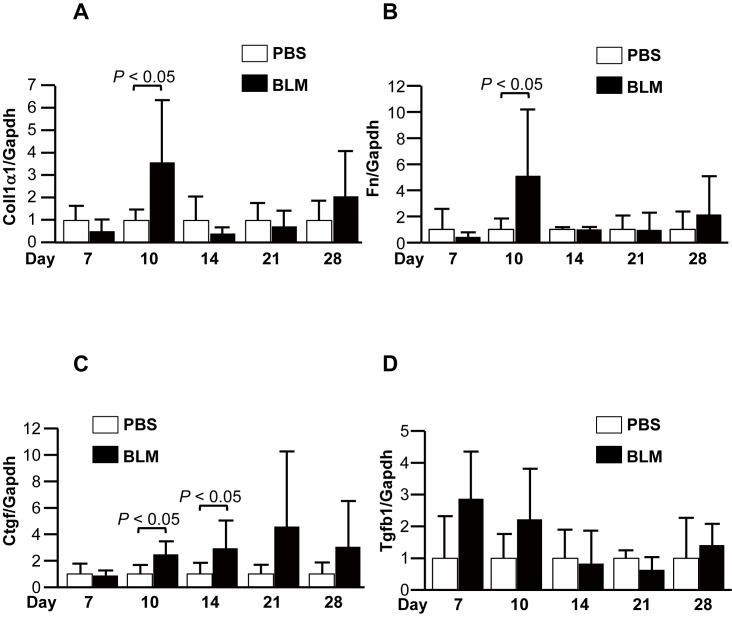
Expression levels of fibrosis-related genes. Mice treated with BLM (60 U/kg) were sacrificed on days 7, 10, 14, 21, and 28. Expression levels of fibrosis-related genes were measured in skin tissues on the indicated days. (A) Coll1α1mRNA levels. (B) Fibronectin mRNA levels. (C) Ctgf mRNA levels. (D) Tgfb1 mRNA levels.

We further analyzed fibrosis-related gene expression in back skin tissues at each time point. qRT-PCR analysis showed that the expression levels of Coll1α1, Fn, and Ctgf were significantly increased in the skin from BLM-treated mice compared with those from control mice on day 10 (Fig 4A–4D). The upregulation of these genes paralleled increased hydroxyproline levels and dermal thickness. Although expression of Coll1α1 and Fn decreased on days 14 through 28, the increased expression of Ctgf was sustained through day 28. Further, Tgfb1 expression levels increased on days 7 and 10, but the differences did not reach statistically significant levels. These results indicated that a higher dose of BLM is required to induce skin fibrosis, but that a lower dose of BLM is sufficient to reduce the thickness of the subcutaneous adipose layer.

### *Ex vivo* induction of fibrosis in human skin treated with BLM

**Fig 5:**
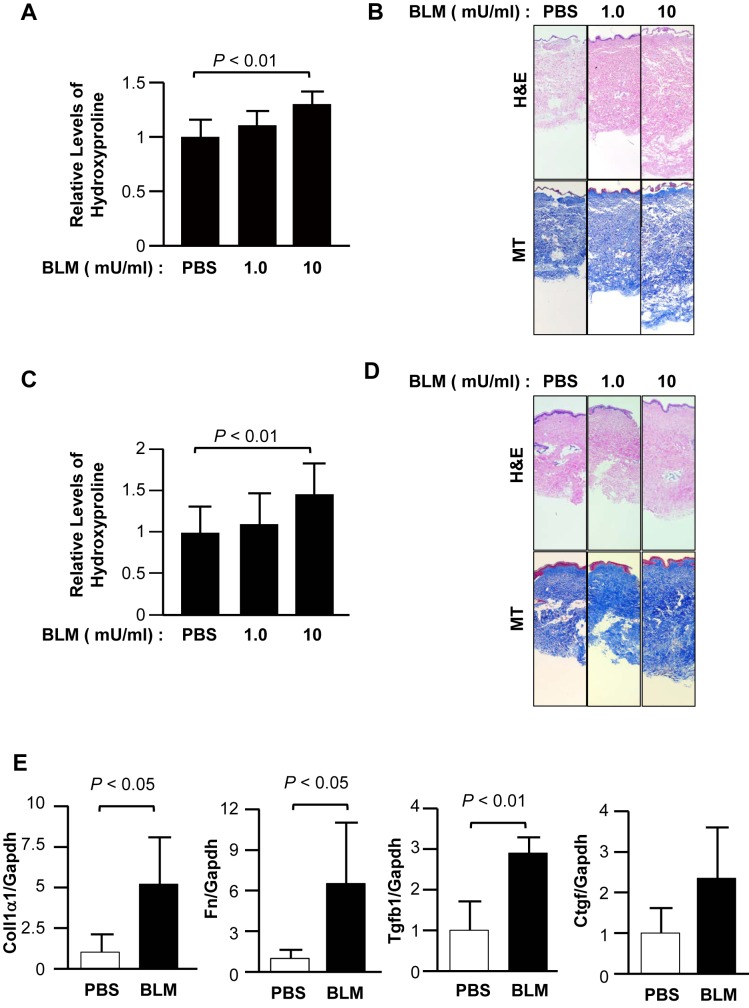
BLM-induced fibrosis in human skin *ex vivo*. Human skin from three different donors was injected with, or immersed in, media containing BLM (1 or 10 mU/ml) and maintained in culture for one week. (A) The amount of collagen in skin injected with BLM was quantified using hydroxyproline assay. (B) Representative images of H&E (upper)–and MT (lower)–stained sections of BLM–injected human skin (original magnification ×25). (C) The amount of collagen in skin immersed in BLM was quantified using hydroxyproline assay. (D) Representative images of H&E (upper)–and MT (lower)–stained sections of BLM–immersed human skin (original magnification ×25). (E) Expression levels of fibrosis-related genes were measured in human skin treated with 10 mU/ml BLM for 48 hours.

To extend findings from the *in vivo* experiments and confirm their relevance to human tissue, we conducted *ex vivo* experiments using human skin maintained in organ culture. We first examined the effect of BLM intradermally injected into human skin. BLM significantly increased the levels of hydroxyproline and dermal thickness in a dose-dependent manner 7 days after injection (Fig 5A and 5B). We next evaluated the effect of immersing skin tissue in media containing BLM. BLM had a similar effect on skin punches immersed in media containing the fibrotic agent (Fig 5C and 5D) for the same duration. We further analyzed fibrosis-related gene expression in human skin treated with BLM at a concentration of 10 mU/ml for 48 hours. qRT-PCR analysis revealed that the expression levels of Coll1α1, Fn, and Tgfb1 were significantly increased in BLM-treated skin compared with control skin (Fig 5E). Although Ctgf mRNA levels also increased from 100% to 360%, the difference did not reach statistical significance. These observations suggest that BLM induces fibrosis in human skin, and similarly to mouse skin, higher doses of BLM are more effective at inducing significant increases in collagen content, expression of fibrosis-associated genes, and ultimately fibrosis.

## Discussion

We show that systemic delivery of BLM via continuous diffusion from subcutaneously implanted osmotic minipumps more closely mimics fibrosis characteristic of human SSc than models in which skin fibrosis is induced when BLM is administered via daily subcutaneous injections or lung fibrosis is induced using intratracheal or intraoral administration of BLM [9]. This model has several advantages for the functional analysis of fibrosis. First, systemic delivery of BLM can cause fibrosis of the skin, lungs, and other internal organs [11], reflecting the systemic nature of the human disease. Second, weight loss and mortality are reduced compared to the more traditional models (data not shown). Third, to induce dermal fibrosis, there is no need for daily repeated injections of BLM which can be laborious. Finally, the BLM pump model may provide the opportunity to examine the effects of potential therapies on fibrosis of the skin and lung simultaneously. However, the mouse strain, dosage of BLM, administration period, and additional important features differ from one report to the next. It is well known that various factors such as the route of administration, mouse strains, and gender influence the degrees of sensitivity in response to BLM-induced fibrosis. In fact, C57BL/6 male mice are more susceptible to dermal fibrosis than female mice using the injection model [19]. Similarly, Balb/C mice develop more severe dermal fibrosis than C57BL/6 and DBA/2 mice [19]. C57BL/6 mice are also more susceptible to induction of pulmonary fibrosis, DBA/2 and Swiss mice are intermediate responders, whereas BALB/c are resistant to lung fibrosis [20].

In this study, the skin and lung fibrosis induced using the pump model were investigated in the context of one mouse strain. Our results revealed that lung fibrosis was dose-dependently increased following BLM treatment, while dermal fibrosis increased in a dose-dependent and transient manner. Dermal thickness and the amount of collagen in skin were significantly increased 10 days post administration of high dose BLM. These findings were supported by changes in expression levels of fibrosis-related genes. However, by day 21, dermal fibrosis had resolved and both collagen content and the expression of fibrosis-related genes had reverted to normal levels. These results indicate that in C57BL/6J mice, lung fibrosis progresses even after the removal of the triggering agent while dermal fibrosis resolves once BLM exposure is terminated. This is in contrast to other reports showing that dermal fibrosis is sustained after the removal of BLM [11–14]. A caveat in comparing dermal thickness between our work and these previous reports is that the distance between the site used to measure dermal thickness and the position of the outlet of the pump that delivers BLM is not clearly stated in previous publications.

A topic of growing interest in studies of skin fibrosis in scleroderma is the relationship between subcutaneous fat and dermal fibrosis. Both in scleroderma patients and mice treated with BLM, dermal fibrosis and thinning of the subcutaneous adipose layer occur [11, 21]. In our studies, we observe that thinning of the subcutaneous adipose layer precedes the thickening of the dermis and is slower to resolve. These observations suggest that the thinning of the adipose layer may contribute to the thickening of the dermis, possibly through a decrease in adipokine production or through an alteration in the ratio of expression of the adipokines adiponectin and leptin. The concept that the thinning of the adipose layer is primary and the thickening of the dermis is secondary is further supported by our observation that at a low dose of BLM, thinning of the adipose layer still occurs although no thickening of the dermis is observed.

There are several differences that can potentially explain the discrepancy in kinetics of dermal fibrosis. One notable difference is the source of BLM. BLM purchased from different manufacturers may not have identical ingredients or identical activity levels. In fact, the same dose of BLM obtained from different manufacturers results in different mortality rates and thus toxicity in the same strain of mice (data not shown). Another important consideration that might explain the different effects of BLM is the choice of mouse strains. C57BL/6 mice are commonly used for modeling diseases and for the generation of genetically engineered mice. However, several C57BL/6 substrains exist, and these are genetically and phenotypically different. Each vendor has maintained their independent breeding stocks for years, leading to genetic differences accumulating. Furthermore, it has been reported that a chromosome 11 mutation in a strain of C57BL/6 mice was detected in stocks maintained by one commercial vendor [15]. This unexpected mutation could result in differences in immune responses. These findings thus suggest that the results of studies using this strain of C57BL/6 mice need to be re-evaluated to ensure that the reported mutation was not responsible for the observed changes. The fact that different investigative groups have used mice from different vendors and even different strains of mice to report data on BLM-induced fibrosis suggests that differences in the kinetics or dose-dependent responses may be influenced by genetic backgrounds. As summarized in Table 1, Liang *et al* showed that continuous administration of BLM (150 mg/kg) via minipumps for 4 weeks to C57BL/6 mice resulted in dermal fibrosis on day 28 [12]. On the other hand, Lemaire *et al* showed that dermal fibrosis in C57BL/6 mice was induced on day 28 even though administration of the BLM (150 mg/kg) was discontinued on day 7 [13]. Lee et al [14] showed that dermal fibrosis can be sustained long after the removal of BLM using CD1 mice.

**Table 1:** Summary of previous reports describing dermal and pulmonary fibrosis using the pump model.

	Mouse Strain	Mouse Vendor	Age	Sex	Amount of BLM	BLM vendor	Duration of BLM	Duration of Experiment	Dermal fibrosis	Lung fibrosis
Lemaire et al [13]	C57BL/6	Harland	6–10 weeks	N/A	100 mg/kg	No data	1 week	28 days	Day 28	No data
Liang et al [12]	C57BL/6	Chinese Academy of Science	6–8 weeks	N/A	150 mg/kg	Nippon Kayaku (Japan)	4 weeks	7, 14, 21, 28 days	Day 28	Day21, 28
Lee et al [14]	CD1	Charles River	10 weeks	Male	100 U/kg	Teva Parenteral Medicines (USA)	1 week	10, 21, 28 days	Day 21, 28	No data
Lee et al [11]	CD1	Charles River	10 weeks	Male	100 U/kg	Teva Parenteral Medicines (USA)	1 week	35 days	Day 35	Day 21, 28, 35

We have previously shown that a single injection of TGF-β induced dermal fibrosis in human skin *ex vivo* [18]. We now show that injection of BLM significantly increased the levels of hydroxyproline, similarly to the effects seen using TGF-β. Moreover, immersing skin in medium containing BLM is more effective at inducing dermal fibrosis than intradermal BLM injections. In this regard, BLM treatment using the immersion method may be an attractive tool for the functional analysis of skin fibrosis in human, or potentially mouse, skin in organ culture.

In conclusion, our findings show that dermal fibrosis in C57BL/6J mice using the pump model can be sustained for 10 days using a higher dose of BLM while lung fibrosis progresses in these mice even after discontinuing BLM administration. The BLM pump model is a powerful tool for the functional analysis of systemic fibrosis and the testing of potential therapies. However, the choice of mouse strains, duration of BLM administration and dose must be carefully considered when using this model.

## Supporting information

S1 Fig Time-dependent changes in inflammatory cell infiltration in mouse lung using the pump model. Representative images of H&E (upper)–and MT (lower)–stained sections of control and BLM–treated lung tissues (original magnification ×25 (left) and ×400 (right)).
(PDF)

S2 Fig The effect of BLM on abdominal skin fibrosis. (A) Dermal thickness was measured in abdominal skin treated with BLM (1 U/kg). (B) Dermal thickness was measured in abdominal skin treated with BLM (60 U/kg).
(PDF)
