# Supplementary figures and images for: Optimization of a murine and human tissue model to recapitulate dermal and pulmonary features of systemic sclerosis
Source: PLoS One. 2017 Jun 26;12(6):e0179917. doi: 10.1371/journal.pone.0179917 (PMC5484495; doi:10.1371/journal.pone.0179917)

**PBS**

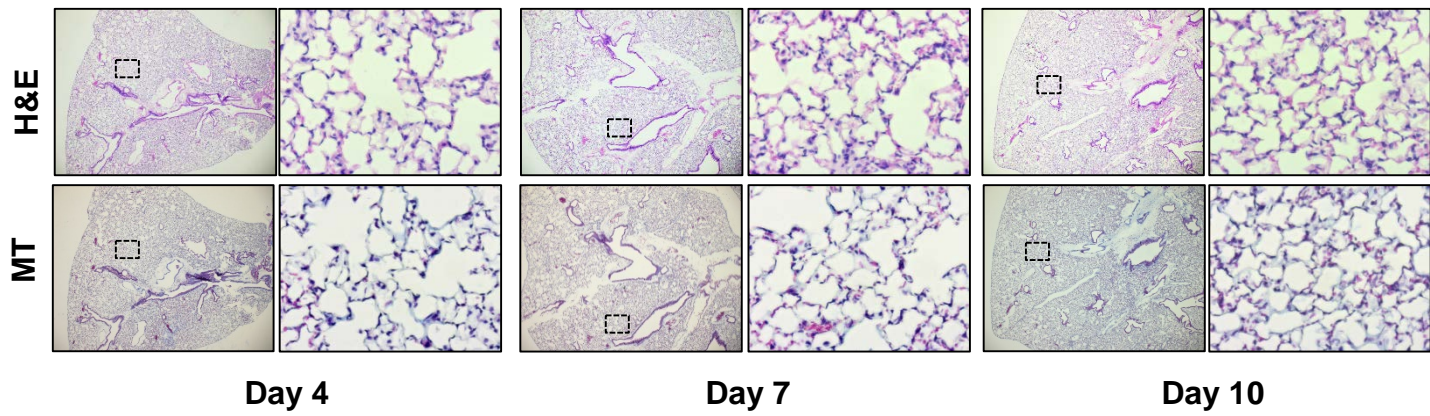

**BLM (1.0 U/kg)**

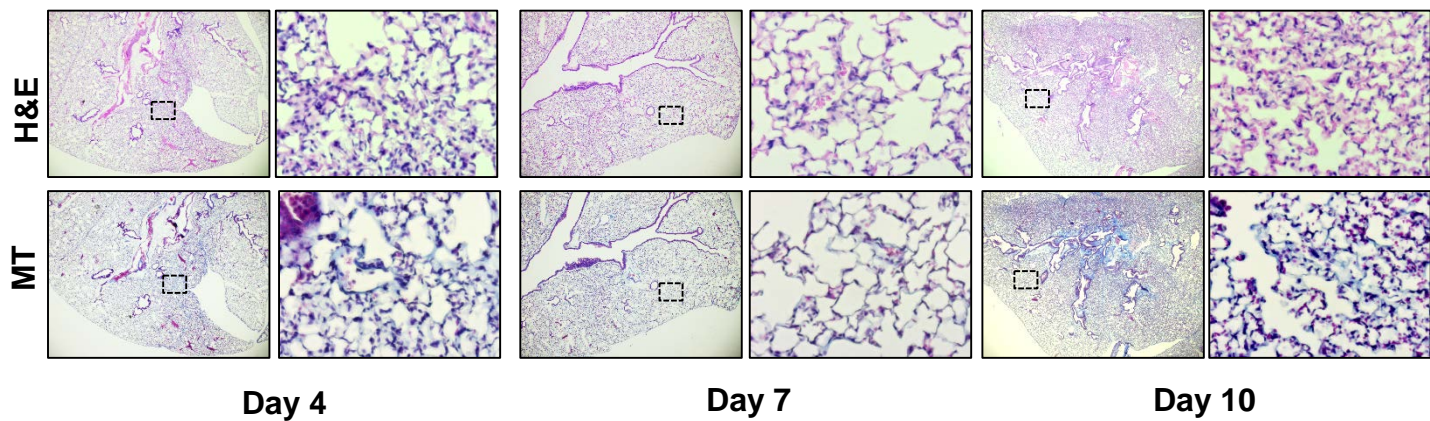

**BLM (60 U/kg)**

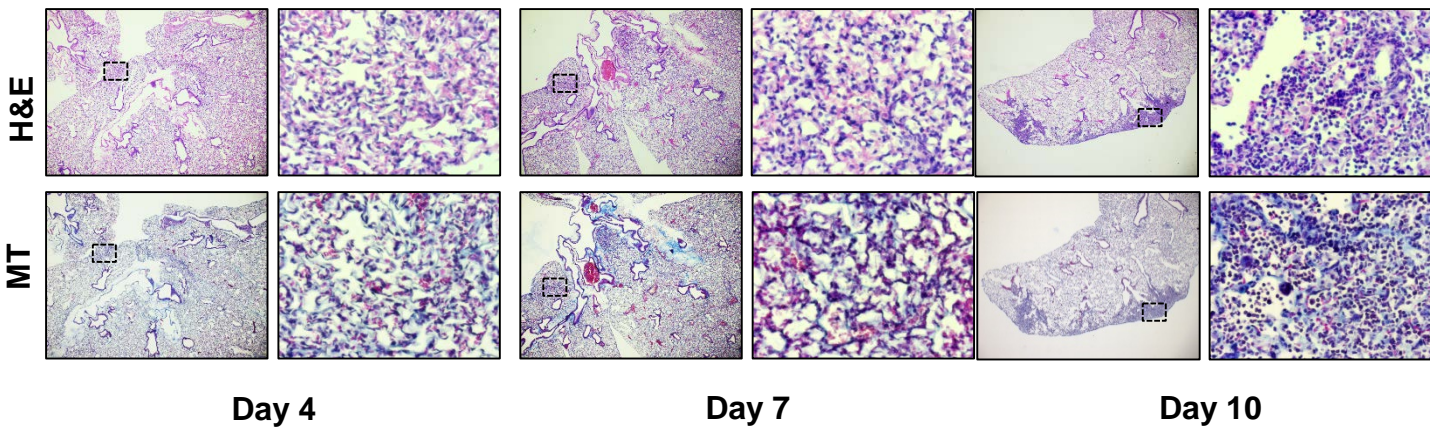

S1 Fig

Supplement: S1 Fig — Representative images of H&E (upper)–and MT (lower)–stained sections of control and BLM–treated lung tissues (original magnification ×25 (left) and ×400 (right)). (PDF) [file pone.0179917.s001.pdf]

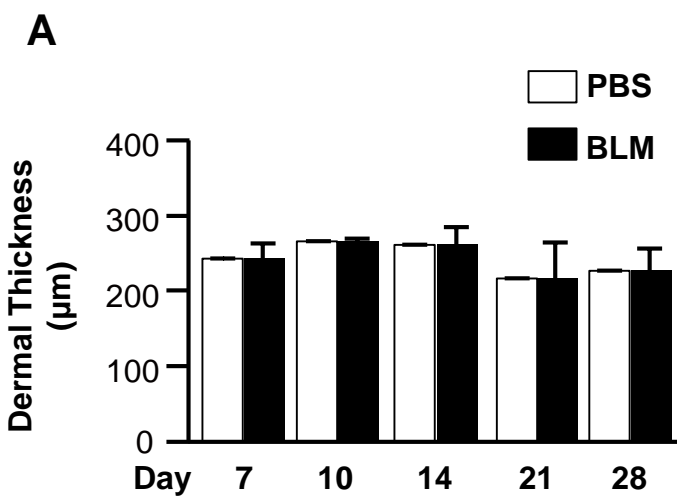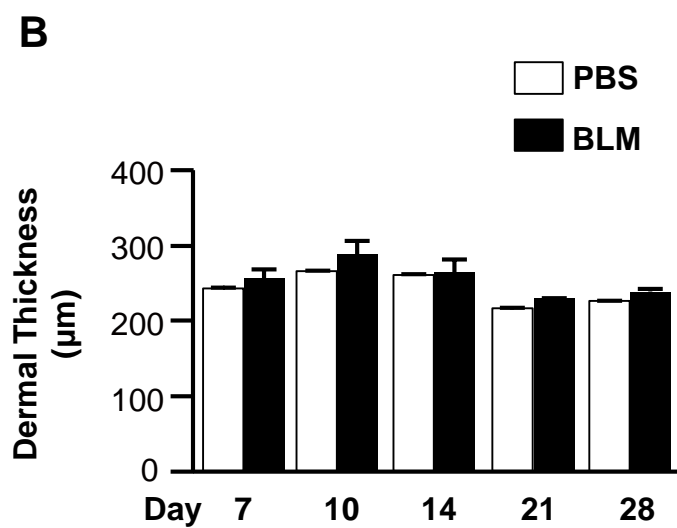

Supplement: S2 Fig — (A) Dermal thickness was measured in abdominal skin treated with BLM (1 U/kg). (B) Dermal thickness was measured in abdominal skin treated with BLM (60 U/kg). (PDF) [file pone.0179917.s002.pdf]
